# Occult Pancreatic Neuroendocrine Tumor Presenting as Carcinoid Syndrome

**DOI:** 10.7759/cureus.62234

**Published:** 2024-06-12

**Authors:** Don Phung, Umair Khizer, Mutahir Farhan, Bashar Saad

**Affiliations:** 1 Internal Medicine, University of California Riverside School of Medicine, Riverside, USA; 2 Internal Medicine, University of California Riverside, Riverside, USA; 3 Endocrinology, Diabetes and Metabolism, University of California Riverside School of Medicine, Riverside, USA

**Keywords:** gallium ga 68 dotatate, mri, ct, pet scan, endocrinology, neuroendocrine tumor, carcinoid syndrome, pancreatic neuroendocrine tumor

## Abstract

We present a case of a 50-year-old male who initially presented to the clinic with complaints of palpitations, shortness of breath, dizziness, night sweats, headaches with associated intermittent episodes of diarrhea, episodes of flushing, and rash on the upper body. Laboratory testing revealed elevated chromogranin A levels. Initial imaging with computed tomography (CT) of the abdomen and pelvis with contrast was negative for any lesions. However, due to his clinical presentation and high suspicion of a neuroendocrine tumor (NET), a positron emission tomography-CT (PET-CT) scan with Gallium 68-DOTATATE was obtained, confirming and localizing his NET in the neck of the pancreas and the liver. Following confirmation and localization of his tumor, he was referred for surgical evaluation and treatment. Pancreatic neuroendocrine tumors are rare and difficult to diagnose, highlighted by unsuccessful initial efforts to localize and confirm the tumor. This case underscores the importance of clinical suspicion and acumen in diagnosing neuroendocrine tumors. Upcoming imaging modalities of PET-CT scans provide promising avenues to uncover neuroendocrine tumors.

## Introduction

Pancreatic neuroendocrine tumors (NETs) are rare tumors that arise in neuroendocrine cells of the pancreas. These tumors can secrete hormones such as insulin, glucagon, and vasoactive intestinal polypeptide (VIP). Neuroendocrine tumors are classified as functioning or non-functioning based on the clinical presentation being consistent with the production of these hormones. Functioning tumors produce and secrete a wide array of hormones that result in symptomatic clinical presentations. Nonfunctioning neuroendocrine tumors either do not produce hormones or produce hormones that do not manifest in clinical symptoms. The wide array of possible peptide hormones results in variable clinical presentations such as seen in insulinoma, glucagonoma, VIPoma, Zollinger-Ellison syndrome, and carcinoid syndrome. Pancreatic NETs are typically found in middle-aged patients (average age of 40 to 60) with estimated incidence rates of <1 case per 100,000 individuals per year [1,2]. Carcinoid syndrome refers to symptoms such as flushing, wheezing, diarrhea, malabsorption, cardiac symptoms (right-sided valvular dysfunction), and fatigue that occur due to the release of hormones from well-differentiated NETs that have metastasized to the liver [3].

## Case presentation

A 50-year-old male with a past medical history of chronic kidney disease stage 3, heart failure with reduced ejection fraction (HFrEF) of < 40%, essential hypertension, previous myocardial infarction, previous cardiac arrest, and gastroesophageal reflux disease presented to the endocrinology clinic with complaints of palpitations, shortness of breath, dizziness, and sweating that began a year ago. His symptoms initially started with shortness of breath at rest and episodic non-exertional chest pain. Over the course of a year, his symptoms continued to progress to dizziness with standing, episodes of nighttime sweating, and daily generalized headaches, along with episodes of intermittent vomiting with watery diarrhea three to four times per week. He noted episodes of flushing with an erythematous rash in the upper trunk and bilateral arms during this period. Given that his symptoms were progressing, he sought care with his primary care physician and was referred to endocrinology for further evaluation. His family history included heart disease and prostate cancer in his father. His social history was unremarkable. He did not report any surgeries.

His vital signs showed tachycardia with a heart rate of 103 beats per minute (BPM) but were otherwise unremarkable. Physical examination revealed an obese male with clear lung sounds and regular abdominal examination with a soft, non-tender abdomen without any palpable masses. Laboratory testing was ordered, and values are summarized in Table 1. Chromogranin A was ordered due to high suspicion for NET, and it was found to be elevated at 6.401 µmol/L and remained elevated upon repeat testing (repeat levels obtained one month after) at 3.614 µmol/L (normal range 0.0-0.577 µmol/L). The patient remained symptomatic from when the labs were ordered until the repeat testing was done.

**Table 1:** Laboratory values

Lab	Result	Normal Range
Serum Metanephrine	328.2 pmol/L	0.0-1,283.0 pmol/L
Serum Normetanephrine	85.11 pmol/L	0.0- 520.17 pmol/L
Serum Aldosterone	0.443 nmol/L	0.083-0.969 nmol/L
Plasma Renin	5.1 mcg/mL/hr	0.25-5.82 mcg/L/hr
Free T4	14.3 nmol/L	10.57-22.83 nmol/L
Thyroid Stimulating Hormone (TSH)	1.650 mIU/L	0.450-4.50 mIU/L
Parathyroid Hormone Intact	35 ng/L	15-65 ng/L
Ionized Calcium	1.225 mmol/L	1.125-1.400 mmol/L
Chromogranin A	6.401 µmol/L	0.0-0.577 µmol/L
Chromogranin A (Repeated)	3.614 µmol/L	0.0-0.577 µmol/L
Serum Serotonin	0.10 μmol/L	0.18-1.18 μmol/L
5-HIAA (Hydroxy Indoleacetic Acid) Urine	26.52 μmol/L	10.92-40.04 μmol/L
5-HIAA (Hydroxy Indoleacetic Acid) Urine 24 hours	40.04 μmol/24 hr	0.0-77.48 μmol/24 hr
Histamine Urine	62.98 nmol/L	26.99-584.81 nmol/L
Histamine Urine 24 Hours	98.97 nmol/24hr	0-584.81 nmol/24hr

**Figure 1:**
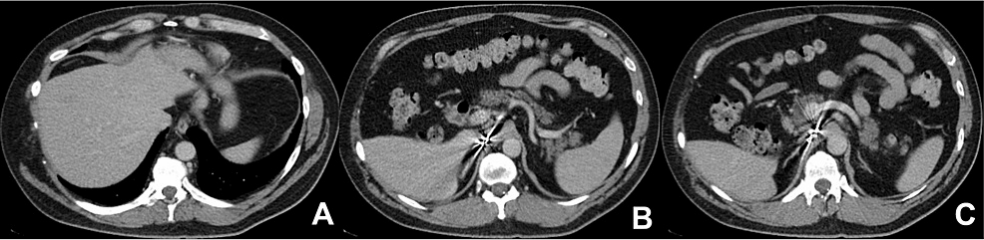
Initial CT of the abdomen and pelvis with contrast without any visible liver or pancreatic lesions

**Figure 2:**
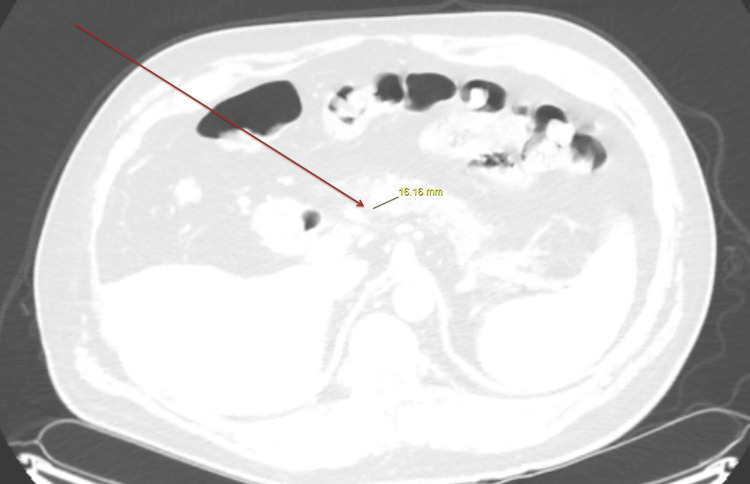
Ga-68 PET-CT with 16-mm hypermetabolic, hyper-vascular lesion in the pancreatic neck-body junction representing pancreatic neuroendocrine tumor

**Figure 3:**
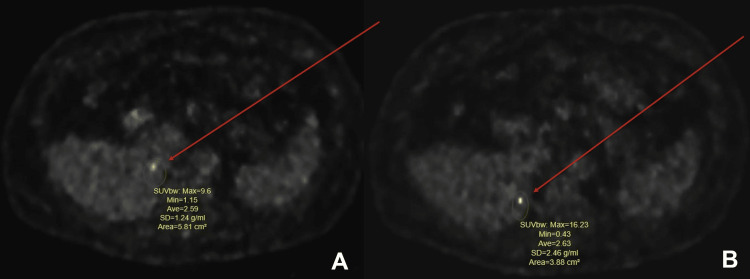
Ga-68 PET-CT with images of two hypermetabolic foci in the medial aspect of the lower right lobe of the liver, representing metastatic liver lesions

Initial imaging with computed tomography (CT) of the abdomen and pelvis with intravenous contrast was ordered to evaluate and localize any possible neuroendocrine tumor; however, the results did not show any abnormalities (Figure 1). Due to continued suspicion of a neuroendocrine tumor, a subsequent Gallium-68 DOTATATE PET-CT was ordered. The PET-CT scan confirmed and localized his NET, revealing a hypermetabolic and hypervascular 1.6-centimeter lesion in the neck of the pancreas and two foci in the right lower lobe of the liver (Figures 2-3). Following confirmation and localization of his tumor, he was then referred for surgical evaluation and treatment. The patient underwent the removal of his neuroendocrine tumor as well as the metastases to the liver with a resolution of his symptoms.

## Discussion

Carcinoid syndrome is a variety of symptoms caused by neuroendocrine tumors, typically manifesting with chronic watery diarrhea and flushing from serotonin and vasoactive peptide products in the systemic circulation [3-5]. Pancreatic NETs are rare entities occurring in 1/100,000 people per year, with only approximately 10% of these NETs resulting in carcinoid syndrome [1,2]. The clinical presentation of carcinoid syndrome results from the secretion of biologically active peptides and amines from NETs into the systemic circulation, typically from the tumor's metastasis to the liver.

Biochemical testing to confirm the diagnosis of suggestive symptoms typically includes 24 urinary excretion measurements of 5-HIAA (5-hydroxy indoleacetic acid, the end product of serotonin metabolism), plasma 5-HIAA measurements, and chromogranin concentrations. Tumor localization and staging are determined by a CT scan (multiphasic contrast-enhanced CT) or MRI [6-8].

In our described case, the patient presented with symptoms suggestive of carcinoid syndrome. The initial biochemical testing revealed elevated levels of chromogranin A. Initial tumor localization through imaging with CT of the abdomen and pelvis with intravenous contrast did not reveal any masses. It is possible that multiphasic sequencing would have enhanced our patient's detection of pancreatic NET and liver metastasis, which the subsequent PET scan uncovered. Magnetic resonance imaging (MRI) and somatostatin receptor-based imaging techniques represent alternative modalities for detecting and localizing NETs. Multiphasic MRI represents a sensitive imaging modality for detecting NETs and metastatic liver lesions [9].

Somatostatin receptor scintigraphy (SRS) represents a form of tumor localization and can also predict clinical response to therapy with somatostatin analog treatment (octreotide, lanreotide). NETs, including nonfunction NETs, have high concentrations of somatostatin receptors and uptake of radiolabeled somatostatin analog and positron emission tomography (PET)-based radionuclides.

More recently, PET-based tracers have emerged (68-DOTATATE, 68-Ga DOTATOC, Cu-64 DOTATATE). In combination, PET and CT scanning have helped improve the detection and staging of NETs, especially of small lesions [10,11]. Clinical suspicion remains highly critical in the diagnosis of NETs. New imaging modalities, such as PET-CT scans, present promising avenues of confirmation and diagnosis, especially in small and occult tumors [11]. Our case highlighted the importance of clinical examination and evolving imaging modalities.

A thorough history, with detailed questioning regarding possible symptoms in conjunction with a comprehensive physical examination and clinical suspicion, helped diagnose the neuroendocrine tumor. Upcoming PET-based tracers offer a promising avenue in the localization and confirmation of obscure pancreatic neuroendocrine tumors. The patient presented with clinically suspicious lesions, but initial testing with a CT scan was negative and required further Gallium 68-DOTATATE PET-CT to uncover pancreatic NET and metastasis to the liver.

## Conclusions

We present a case of a middle-aged male who initially presented to the clinic with complaints of palpitations, night sweats, headaches, diarrhea, and flushing suspicious of carcinoid syndrome. Pancreatic neuroendocrine tumors are challenging to diagnose, requiring astute clinical examination and high clinical suspicion for diagnosis. They can be small in size and often difficult to localize on imaging. Our case illustrates the rarity and difficulty of diagnosing neuroendocrine tumors. Initial imaging with a CT scan of the abdomen in an attempt to localize and stage the tumor was unsuccessful, requiring advanced imaging with Gallium-68 DOTATATE in order to diagnose the neuroendocrine tumor. Clinical examination and upcoming imaging modalities like PET-based tracers such as Gallium-68 DOTATATE PET-CT scan provide the optimal diagnostic opportunity to uncover these obscure tumors.
